# Improving CD3 bispecific antibody therapy in solid tumors using combination strategies

**DOI:** 10.3389/fonc.2025.1548446

**Published:** 2025-02-10

**Authors:** Katy Lloyd, Jim Middelburg, Vitalijs Ovcinnikovs, Nora Pencheva, Kristel Kemper, Thorbald van Hall

**Affiliations:** ^1^ Genmab B.V., Utrecht, Netherlands; ^2^ Department of Medical Oncology, Oncode Institute, Leiden University Medical Center, Leiden, Netherlands

**Keywords:** antibody, CD3, bispecific Ab, cancer, vaccination

## Abstract

CD3 bispecific antibodies (bsAbs) are emerging as an important treatment option in the arsenal of oncologists. There are numerous FDA-approved CD3 bsAbs for both hematological and solid tumors. Despite these recent advances, the success of CD3 bsAbs in solid cancer has been hampered by hurdles like limited intratumoral T cell numbers, immunosuppressive tumor microenvironments (TME), and poor memory T-cell induction. Furthermore, tumor surface antigen selection for an optimal therapeutic window and acceptable collateral damage to normal tissues is challenging. In this review, we discuss recent research investigating combination approaches aimed at improving CD3 bsAb efficacy in solid cancer.

## Introduction

**Figure 1:**
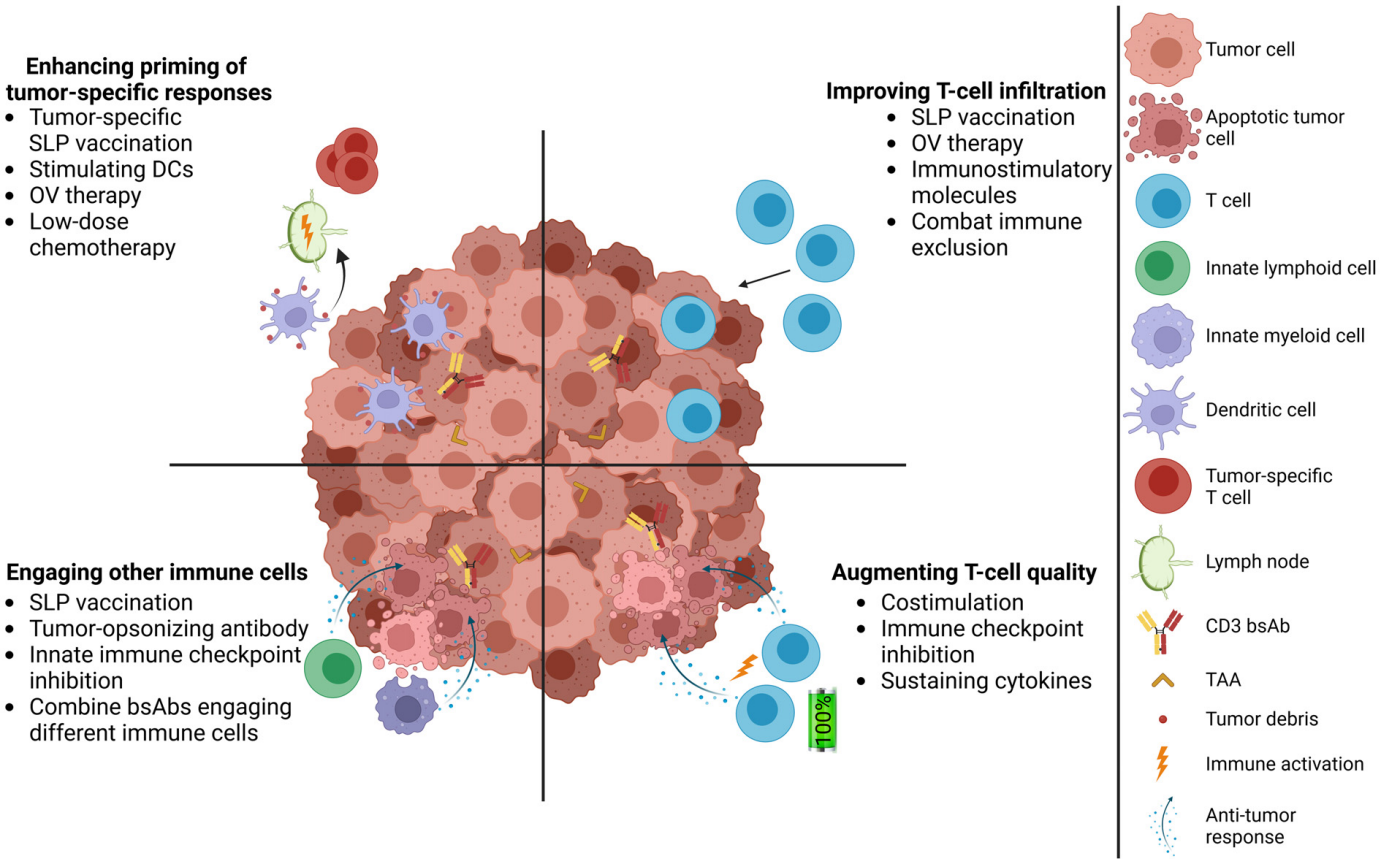
Complementing CD3 bsAb therapy with cancer vaccines in solid tumors. Addressing multiple hurdles of CD3 bsAb therapies, including promoting T-cell infiltration, increasing the quality of intratumoral T cells, engaging innate immune cells in the TME, and induction of tumor-specific T-cell responses. TAA, tumor-associated antigen; SLP, synthetic long peptide; DC, dendritic cell; OV, oncolytic virus.

CD3 bsAbs bind distinct antigens with each Fab arm, allowing them to simultaneously engage CD3 on a T cell and a tumor-associated antigen (TAA) expressed on tumor cells (1). CD3 bsAb-mediated cross-linking of these two cell types facilitates immunological synapse formation and subsequent T-cell activation, potentiating tumor cell kill via the secretion of cytolytic components and inflammatory cytokines (2, 3). By engaging CD3, bsAbs can recruit all available T cells to mediate tumor kill, regardless of their cognate specificity. As of December 2024, there are seven CD3 bsAbs with US Food and Drug Administration (FDA) approval for treatment of hematological cancers (**Table 1**) (4–7, 9–11). To date, the success in solid tumors remains limited, with two FDA approvals for Tebentafusp^®^ (advanced uveal melanoma) (12) and Tarlatamab^®^ (extensive-stage small cell lung cancer) (13). Despite these advances, there are still several hurdles for CD3 bsAb therapy in solid tumors (14) (**Figure 1**). Choosing a suitable tumor surface antigen is critical for clinical success, with consequences for an optimal therapeutic window and acceptable collateral damage to normal tissues. Additional hurdles include limited numbers of intratumoral T cells, an immunosuppressive tumor microenvironment (TME), and poor memory T-cell induction. We recently investigated several combination approaches to address these hurdles (15, 16), which are highlighted here within.

**Table 1:** FDA-approved CD3 bispecific therapies.

Drug name	Company	Targets	Format	Cancer type	Indication	FDA-approval	Ref
Blinatumomab	Amgen	CD3xCD19	BiTE	Hematological	Acute Lymphoblastic Leukemia	Dec 2014	(4)
Teclistamab	Janssen	CD3xBCMA	DuoBody	Hematological	Multiple Myeloma	Oct 2022	(5)
Mosunetuzumab	Genentech	CD3xCD20	Knob-in-hole bsAb (1 + 1)	Hematological	Follicular Lymphoma	Dec 2022	(6)
Epcoritamab	AbbVie & Genmab	CD3xCD20	DuoBody	Hematological	Diffuse Large B Cell Lymphoma, Follicular Lymphoma	May 2023 Jun 2024	(7) (8)
Glofitamab	Roche	CD3xCD20	Knob-in-hole bsAb (2 + 1)	Hematological	Large B Cell Lymphoma	Jun 2023	(9)
Talquetamab	Janssen	CD3xGPRC5D	DuoBody	Hematological	Multiple Myeloma	Aug 2023	(10)
Elranatamab	Pfizer	CD3xBCMA	IgG1/IgG2 bsAb	Hematological	Multiple Myeloma	Aug 2023	(11)
Tebentafusp	Immunocore	CD3xHLA-A*0201/ gp100	ImmTAC	Solid	Uveal melanoma	Jan 2022	(12)
Tarlatamab	Amgen	CD3xDLL3	BiTE-Fc	Solid	Small Cell Lung Cancer	May 2024	(13)

## Improving T-cell infiltration in solid tumors

In contrast to hematological malignancies that are surrounded by T cells, solid cancers often harbor TMEs that contain limited T-cell numbers. This feature might be associated with impaired CD3 bsAb therapeutic efficacy in preclinical models (17), which is most prominent in immunologically “cold” tumors exhibiting poor T-cell infiltration (18). We recently showed in CXCR3 knock-out mice that CD3 bsAb antitumor activity in an immunologically “cold” tumor model was dependent on the influx of T cells (15, 19–22), supporting comparable preclinical findings that higher intratumoral T-cell numbers, either present at baseline or through therapy-induced influx, improve CD3 bsAb outcomes (20, 23).

We thus examined strategies that would boost intratumoral T-cell frequencies aiming to improve CD3 bsAb efficacy in immunology “cold” tumors. We reached significantly enhanced tumor eradication when T-cell responses were mounted via tumor nonspecific vaccines prior to CD3 bsAb (15). Vaccine adjuvants IL-2 and TLR7 agonist imiquimod, or TLR9 agonist CpG, were used to ensure sufficient co-stimulation in the lymphatics, enhancing T-cell priming and functionality. We observed that vaccination induced homing of activated CD8^+^ T cells towards the tumor rim and that subsequent CD3 bsAb administration further transformed T cells into potent effectors and enabled their deep infiltration into the tumor core (15). This process shares similarities with the proposed two-stage tumor-specific CD8^+^ T-cell activation previously reported (24), whereby initial activation in tumor-draining lymph nodes is followed by subsequent cytotoxic effector program acquisition after target recognition in the TME. We showed that tumor-specific or -nonspecific vaccination in combination with CD3 bsAb promoted a broadly inflamed TME, delayed tumor outgrowth and improved survival in mice (15). The irrelevance of vaccine antigen specificity to primary survival benefit is likely explained by unbiased trafficking of vaccine-activated T cells to non-lymphoid tissues, including the tumor, where engagement by the CD3 bsAb occurs regardless of the cognate specificity. Importantly, CD3 bsAb therapy with adjuvants alone did not improve tumor control in “cold” tumors, highlighting the necessity of an immunogenic antigen to generate substantial CD8 T-cell expansion and influx, resulting in significant survival benefits. Interestingly, combining chimeric antigen receptor (CAR) T-cell therapies with cognate antigen vaccination have shown superior engraftment, polyfunctionality and antitumor activity compared to non-vaccinated controls (25–27). Thus, vaccination could offer a relatively cheap “off-the-shelf” approach to systemically boost T cells during T-cell targeting therapies.

An alternative approach based on the same principle utilized oncolytic viruses (OV) to enhance CD3 bsAb therapy. Selective viral replication in tumor cells was shown to initiate a localized anti-viral immune response and strong influx of OV-specific T cells that could be subsequently engaged by CD3 bsAb (28). Others have reported similar survival benefits using intratumorally-injected OV constructs encoding for CD3 bsAbs (29), which limits antibody production to the tumor and potentially reduces systemic exposure and toxicities. However, this could result in CD3 bsAb not reaching distal tumors, whereas induction of an anti-viral response against the OV may either terminate bsAb production or, conversely, result in antigen and DAMP release. If both strategies demonstrate comparable clinical efficacy, the vaccination approach may be preferred to OVs, as it would not be limited to easily accessible tumors or by an anti-viral immune response, thereby allowing subsequent booster vaccinations to maintain T-cell influx.

## Improving T-cell functionality in solid tumors

Solid cancers typically feature hostile and immunosuppressive TME, due to factors such as low pH, hypoxia, nutrient deprivation and high concentration of oxygen radicals, which can hamper cytotoxic T-cell effector functions (14). In addition, chronic exposure to antigens leads to exhaustion of T cells and expression of a plethora of inhibitory immune receptors. In the clinic, T-cell functionality also declines with prior rounds of systemic chemotherapy and disease progression, which may pose a challenge for CD3 bsAbs in heavily pre-treated patient populations (30, 31). There is some clinical data that correlate good T-cell functionality with clinical response to CD3 bsAb therapy in solid tumors, suggesting this is a likely contributing factor (32).

As immune checkpoint expression can be induced following CD3 bsAb therapy (33, 34), immune checkpoint inhibitors (ICIs) are frequently tested in combination with CD3 bsAbs, with many ongoing clinical trials for solid tumors (35), aimed at lifting the checkpoint-mediated brake on T-cell activation (**Figure 1**). Multiple preclinical studies show augmented T-cell functionality and therapeutic benefit when combing ICIs and CD3 bsAbs compared to CD3 bsAb monotherapy (36–38). Similarly, there are also indications that combining CAR T-cell therapy with ICIs could improve therapeutic outcomes (39). As we observed enhanced PD-1, NKG2A, CTLA-4, Tim-3 and TIGIT expression on T cells in mice treated with CD3 bsAb and vaccine combinations, suggesting that peripherally-derived T cells are more fit than TME-localized T cells (15), it would be interesting to test if the addition of ICIs could further enhance therapeutic efficacy.

Another strategy for improving T-cell functionality involves combining CD3 bsAbs with agonistic antibodies that trigger costimulatory signals. The concept is supported by second-generation CAR T-cell therapies which feature a costimulatory signaling domain, to provide a “signal 2” to T cells in addition to CD3 signaling (signal 1), resulting in superior T-cell persistence and functionality (40, 41). Preclinical studies have reported therapeutic benefit when combining CD3 bsAbs with agonistic antibodies providing costimulation via either 4-1BB or CD28 (42–44). Likewise, we observed improved tumor control when combining tumor-localized 4-1BB costimulation with CD3 bsAb therapy in mice inoculated with immunologically “cold” tumors (45). Given the boost in T-cell function and upregulation of immune checkpoints after T-cell activation, strategies combining CD3 bsAb with costimulatory signals seem promising for improving therapeutic benefit. However, further research is needed to compare the safety and efficacy of such combinations.

## Tumor-specific responses are crucial for protective memory following CD3 bsAb therapy

CD3 bsAb therapy has displayed impressive response rates in hematological malignancies in the clinic, but relapses are frequently reported (46–48). Tumor heterogeneity or downmodulation of surface expression of the targeted antigen is responsible for a large proportion of relapses, both for CD3 bsAbs and CAR T cells (46–50). While this phenomenon has not yet been described in the clinic for solid tumors, there are similar indications in preclinical studies (28), including ours, where escape of TRP1^-^ cells was observed in end-stage tumors from CD3 bsAb-treated mice (45). To overcome this and decrease immune escape, combinations of multiple CD3 bsAbs or CAR T-cell specificities targeting different tumor antigens are being evaluated (51, 52).

An alternative approach to prevent escape through antigen loss is the induction of endogenous responses against antigen-negative tumor cells (i.e. antigen spread), which may arise after tumor antigen release upon tumor lysis and subsequent presentation by antigen presenting cells (APCs) (53). We previously reported that although CD3 bsAb monotherapy induced tumor-specific T-cell responses, these responses were short-lived and did not install protective memory in the immunologically “cold” B16F10 model (54). We also observed improved primary responses but no protection from secondary B16F10 tumor challenge when combining CD3 bsAb with 4-1BB costimulation, Fc-active tumor-opsonizing antibodies, or tumor-nonspecific vaccines (45).

Combining CD3 bsAb and tumor-specific vaccination enhanced survival against primary tumors and delayed tumor outgrowth upon secondary tumor challenge (45). However, complete protection against tumor rechallenge was not reached, implying there was insufficient antigen spread in the immunologically “cold” tumor model. Therefore, we hypothesized that the expression of a highly immunogenic antigen may be required for functional memory. In the immunologically “hot” MC38 tumor model, tumor antigen spreading occurred independent of the administered treatment. Tumor-specific T cells were detected in all mice, and most mice that had completely eradicated the first tumor were protected from MC38 tumor rechallenge (45). Protection against rechallenge was completely ablated upon CD8^+^ T-cell depletion, highlighting the necessity of tumor-specific endogenous CD8^+^ T-cell responses for long-term memory. There was substantial outgrowth of antigen-negative tumor cells in mice treated with CD3 bsAb monotherapy, but not when CD3 bsAb was combined with vaccination, highlighting the need to strike hard during primary tumor treatment to prevent early immune escape (45).

Our results suggest that “off-the-shelf” T-cell stimulating vaccines can be utilized to enhance CD3 bsAb anti-tumor activity against primary solid tumors. However, preventing relapse requires a level of intrinsic tumor immunogenicity and can be further bolstered by tumor-specific immunization (**Figure 1**). Such combinations could shortly become a reality given the recent success of personalized mRNA cancer vaccines in the clinic (55). Given the local and potent immune activation and concomitant enhanced T-cell infiltration with OVs, these could also serve as an alternative CD3 bsAb combination partner to drive endogenous anti-tumor T-cell responses (56).

In summary, CD3 bsAbs and vaccine combinations improved survival against primary tumors and tumor rechallenge in mice. The immune status of tumors impacted survival benefit, where CD3 bsAb anti-tumor activity in tested immunologically “hot” tumor models benefited from innate activators, whereas “cold” tumor models required provision of an immunogenic tumor antigen. It remains to be determined if combinations with novel therapies such as personalized mRNA vaccines can benefit CD3 bsAb therapy in the clinic.

## Improving CD3 bsAb therapy through engagement of other immune cells

Emerging evidence suggests that a combination of both innate and adaptive immunity is pivotal for complete and durable tumor eradication following immunotherapy (57–60) and understanding immune cell interplay in the TME can thus further improve therapeutic efficacy. Recently, we reported that the CD3 bsAb and vaccine combination increased frequencies of intratumoral CD8^+^ T cells, NK cells, inflammatory macrophages and neutrophils in the B16F10 model (16), supporting previous findings that CD3 bsAb therapy enhances NK-cell and macrophage activation (61). We revealed that CD3 bsAb-activated T cells attracted macrophages into the tumor and skewed them towards a late-stage, pro-inflammatory phenotype (16). These macrophages were crucial for the anti-tumor activity of the CD3 bsAb and vaccination combination, highlighting the importance of a coordinated innate and adaptive immune response for effective tumor control (16). Furthermore, comparable late-stage pro-inflammatory macrophage subsets were identified in human cancers by gene expression analysis (62), which were found to correlate with ICI treatment response in a breast cancer cohort (16, 63). Our findings align with studies demonstrating crucial roles for innate effector cells in immunotherapy (16, 60, 64), supporting the rationale for the so far unexplored combination of innate cell-stimulating therapeutics with CD3 bsAbs. There is a number of novel therapeutic strategies seeking to empower innate responses against cancer, including stimulation of pattern recognition receptors like stimulator of interferon genes (GAS-STING), TLR, and RIG-I-like receptors (RLR), as well as modulation of macrophages and natural killer cells (65). Immune-stimulating antibody conjugates (ISAC) combine therapeutic antibodies and innate-stimulating payloads, such as TLR or STING agonists, to promote inflammatory TMEs (66). Several ISACs have been clinically evaluated, including Bolt Biotherapeutic’s anti-HER2-TLR7/8 (BDC-1001) for HER2-positive solid tumors (NCT04278144) and Takeda’s anti-CCR2-STING (TAK-500) for various solid tumors (NCT05070247). Another way to potentially improve intratumoral T-cell functionality and boost innate effectors during CD3 bsAb therapy is to co-administer sustaining cytokines, similar to strategies using genetically engineered CAR T cells that express cytokines upon activation for improved therapeutic outcomes (67). There are reports showing positive contributions of cytokines to CD3 bsAb therapy preclinically, including combinations with immunomodulating drugs that induce IL-2 (68), Fc-fused IL-15 compounds (69), and bsAb-IL-15 fusions (70). Although the clinical efficacy of innate activators remains to be elucidated, especially in relation to combinations with CD3 bsAb, the recent FDA approval of ImmunityBio’s IL-15 receptor agonist with Bacillus Calmette-Guérin (BCG) vaccination for bladder cancer highlights the potential of next-generation innate stimulators for cancer immunotherapy (71).

Harnessing innate immune cells could greatly potentiate CD3 bsAb efficacy. As one of the safety risks of CD3 bsAb therapy is cytokine release syndrome, it remains to be determined if this is exacerbated by combinations with vaccination and/or innate stimulators. Furthermore, it will be important to consider that innate immune cell engagement could be context-dependent, as engagement of predominantly immunosuppressive immune cells might even lower therapeutic outcomes.

## Remaining questions and path to clinical application

To ensure clinical success of CD3 bsAb and T-cell stimulating vaccine combination therapies, numerous unanswered questions and knowledge gaps must be addressed. For example, deeper understanding on vaccine prerequisites for sufficient T-cell priming prior to CD3 bsAb therapy is needed. Ideally, vaccines should elicit a Th1 response in CD8^+^ T cells. Most marketed childhood and travelers’ vaccines are prophylactic in nature, designed to induce neutralizing Abs, requiring (pre)clinical studies to determine their suitability for inducing Th1 responses supporting CD8^+^ cytolytic T cells. In addition to their wide availability, a key advantage of approved prophylactic vaccines is their well-established safety profiles, facilitating clinical evaluation with an experimental CD3 bsAb agent. T-cell recall responses from previous immunizations could be utilized (**Supplementary Table**), as retrospective analyses suggest beneficial associations between ICI outcomes and earlier Covid-19 or influenza vaccination (72–74). However, prospective randomized clinical trials are required to fully understand if prophylactic tumor non-specific vaccinations can improve immunotherapy efficacy in human cancers. Our murine studies suggest that tumor-specific vaccination may be a more suitable choice, owing to its ability to improve both survival against primary tumor and protection against tumor recurrence (45). Cancer vaccine development has historically faced challenges with limited success (75–77). Recent advances in the field of precision medicine and mRNA-technologies may help overcome the shortcomings of traditional cancer vaccines and potentially revolutionize cancer immunotherapy. Many next-generation cancer vaccines are in clinical trials in combination with ICI (55, 78, 79), and given our preclinical results, it would be of interest to explore their efficacy in combination with CD3 bsAbs.

Further understanding of the role of adjuvants is warranted, as this may affect clinical applicability of the approach. Since we depicted a pivotal role for T-cell and macrophage cooperation in therapeutic response (16), it may be interesting to evaluate which vaccine adjuvants or innate activators are optimal to prime innate cells for contributing to CD3 bsAb efficacy. Recent phase II trial data for patients with HPV-induced abnormal cervical cells showed localized treatment with imiquimod promoted regression of high-grade lesions and significantly increased T-cell infiltration in the cervix (80). The addition of an HPV vaccine had no impact on therapeutic outcome (80), similar to our preclinical observations that tumor-specific vaccination with adjuvants imiquimod and IL-2 was insufficient for tumor control (15). Since CD3 bsAb and tumor-specific vaccine combinations enhanced tumor control in our study (15), combining CD3 bsAb with imiquimod could further improve treatment outcomes for cervical indications.

Another important area of research is the impact of baseline T-cell fitness on treatment outcome. The phenotype and functional status of endogenous circulating T cells at baseline are emerging as important parameters for the clinical activity for T-cell engagers (17, 32, 81, 82). The requirement of baseline T-cell infiltration in tumor nests for therapeutic efficacy is unclear, as shown recently for CD3xCEA in MSS-CRC patients (83). In addition, the two FDA-approved CD3 bsAbs for solid tumors show efficacy in indications that are notorious for being immunologically “cold” and for which ICI therapy is largely ineffective (12, 13). These findings suggest the initial T-cell activation signal may come from a limited number of T cells interacting with tumor cells at the edge of the tumor nests, resulting in a gradient of chemokines summoning circulating T cells to tumor sites, thus promoting T-cell infiltration (83). Since we showed that combining CD3 bsAb with vaccination helped recruit fresh peripheral T cells to the tumor via the CXCR3 axis in mouse models, clinical studies are warranted to evaluate if influx of peripheral cells into the tumor also occurs in humans and how priming may impact T-cell fitness pre- and post-CD3 bsAb administration.

Finally, successful clinical translation will need to enhance CD3 bsAb efficacy whilst maintaining an acceptable safety window. Activation of T cells by vaccination followed by CD3 bsAb, with or without additional innate activators, could drive immune-related adverse events. Furthermore, we need to understand vaccine response kinetics in relation to CD3 bsAb administration and whether there is a preference for either recall or *de novo* response. Rational bsAb design, careful selection of the target antigen, and dose-optimization strategies could mitigate potential dose-limiting toxicities, as well as potentially reinforcing clinical activity of CD3 bsAbs in solid tumors (83–86) (**Figure 1**).

To conclude, we advocate for the clinical translation of T-cell stimulating vaccines during CD3 bsAb therapy for superior efficacy against primary tumors and to potentially drive immunological memory. The combination could offer a versatile and effective means to improve cancer immunotherapy outcomes, which may be applicable to various tumor types, immunotherapies, and vaccine modalities.

## References

[1] Labrijn AF Janmaat ML Reichert JM Parren P. Bispecific antibodies: a mechanistic review of the pipeline. Nat Rev Drug Discovery. (2019) 18:585–608. doi: 10.1038/s41573-019-0028-1 31175342

[2] Roda-Navarro P Álvarez-Vallina L. Understanding the spatial topology of artificial immunological synapses assembled in T cell-redirecting strategies: A major issue in cancer immunotherapy. Front Cell Dev Biol. (2020) 7:370. doi: 10.3389/fcell.2019.00370 31998721 PMC6965029

[3] Trabolsi A Arumov A Schatz JH. T cell-activating bispecific antibodies in cancer therapy. J Immunol. (2019) 203:585–92. doi: 10.4049/jimmunol.1900496 31332079

[4] Kantarjian H Stein A Gökbuget N Fielding AK Schuh AC Ribera JM. Blinatumomab versus chemotherapy for advanced acute lymphoblastic leukemia. N Engl J Med. (2017) 376:836–47. doi: 10.1056/NEJMoa1609783 PMC5881572 28249141

[5] Moreau P Garfall AL van de Donk N Nahi H San-Miguel JF Oriol A. Teclistamab in relapsed or refractory multiple myeloma. N Engl J Med. (2022) 387:495–505. doi: 10.1056/NEJMoa2203478 35661166 PMC10587778

[6] Budde LE Sehn LH Matasar M Schuster SJ Assouline S Giri P. Safety and efficacy of mosunetuzumab, a bispecific antibody, in patients with relapsed or refractory follicular lymphoma: a single-arm, multicentre, phase 2 study. Lancet Oncol. (2022) 23:1055–65. doi: 10.1016/S1470-2045(22)00335-7 35803286

[7] Thieblemont C Phillips T Ghesquieres H Cheah CY Clausen MR Cunningham D. Epcoritamab, a novel, subcutaneous CD3xCD20 bispecific T-cell-engaging antibody, in relapsed or refractory large B-cell lymphoma: dose expansion in a phase I/II trial. J Clin Oncol. (2023) 41:2238–47. doi: 10.1200/JCO.22.01725 PMC10115554 36548927

[8] Vose J Vitolo U Lugtenburg P Chamuleau MED Linton KM Thieblemont C. EPCORE NHL-1 follicular lymphoma (FL) cycle (C) 1 optimization (OPT) cohort: Expanding the clinical utility of epcoritamab in relapsed or refractory (R/R) FL. J Clin Oncol. (2024) 42:7015. doi: 10.1200/JCO.2024.42.16_suppl.7015

[9] Dickinson MJ Carlo-Stella C Morschhauser F Bachy E Corradini P Iacoboni G. Glofitamab for relapsed or refractory diffuse large B-cell lymphoma. N Engl J Med. (2022) 387:2220–31. doi: 10.1056/NEJMoa2206913 36507690

[10] Chari A Minnema MC Berdeja JG Oriol A van de Donk N Rodríguez-Otero P. Talquetamab, a T-cell-redirecting GPRC5D bispecific antibody for multiple myeloma. N Engl J Med. (2022) 387:2232–44. doi: 10.1056/NEJMoa2204591 36507686

[11] Lesokhin AM Tomasson MH Arnulf B Bahlis NJ Miles Prince H Niesvizky R. Elranatamab in relapsed or refractory multiple myeloma: phase 2 MagnetisMM-3 trial results. Nat Med. (2023) 29:2259–67. doi: 10.1038/s41591-023-02528-9 PMC10504075 37582952

[12] Nathan P Hassel JC Rutkowski P Baurain JF Butler MO Schlaak M. Overall survival benefit with tebentafusp in metastatic uveal melanoma. N Engl J Med. (2021) 385:1196–206. doi: 10.1056/NEJMoa2103485 34551229

[13] Ahn MJ Cho BC Felip E Korantzis I Ohashi K Majem M. Tarlatamab for patients with previously treated small-cell lung cancer. N Engl J Med. (2023) 389:2063–75. doi: 10.1056/NEJMoa2307980 37861218

[14] Middelburg J Kemper K Engelberts P Labrijn AF Schuurman J van Hall T. Overcoming challenges for CD3-bispecific antibody therapy in solid tumors. Cancers (Basel). (2021) 13:287. doi: 10.3390/cancers13020287 33466732 PMC7829968

[15] Middelburg J Sluijter M Schaap G Göynük B Lloyd K Ovcinnikovs V. T-cell stimulating vaccines empower CD3 bispecific antibody therapy in solid tumors. Nat Commun. (2024) 15:48. doi: 10.1038/s41467-023-44308-6 38167722 PMC10761684

[16] van Elsas MJ Middelburg J Labrie C Roelands J Schaap G Sluijter M. Immunotherapy-activated T cells recruit and skew late-stage activated M1-like macrophages that are critical for therapeutic efficacy. Cancer Cell. (2024) 42:1032–50.e10. doi: 10.1016/j.ccell.2024.04.011 38759656

[17] Ströhlein MA Lefering R Bulian DR Heiss MM. Relative lymphocyte count is a prognostic parameter in cancer patients with catumaxomab immunotherapy. Med Hypotheses. (2014) 82:295–9. doi: 10.1016/j.mehy.2013.12.014 24411128

[18] Patel D Balderes P Lahiji A Melchior M Ng S Bassi R. Generation and characterization of a therapeutic human antibody to melanoma antigen TYRP1. Hum Antibodies. (2007) 16:127–36. doi: 10.3233/HAB-2007-163-407 18334748

[19] Belmontes B Sawant DV Zhong W Tan H Kaul A Aeffner F. Immunotherapy combinations overcome resistance to bispecific T cell engager treatment in T cell-cold solid tumors. Sci Transl Med. (2021) 13:eabd1524. doi: 10.1126/scitranslmed.abd1524 34433637

[20] Cremasco F Menietti E Speziale D Sam J Sammicheli S Richard M. Cross-linking of T cell to B cell lymphoma by the T cell bispecific antibody CD20-TCB induces IFNγ/CXCL10-dependent peripheral T cell recruitment in humanized murine model. PloS One. (2021) 16:e0241091. doi: 10.1371/journal.pone.0241091 33406104 PMC7787458

[21] Li J Ybarra R Mak J Herault A De Almeida P Arrazate A. IFNgamma-induced chemokines are required for CXCR3-mediated T-cell recruitment and antitumor efficacy of anti-HER2/CD3 bispecific antibody. Clin Cancer Res. (2018) 24:6447–58. doi: 10.1158/1078-0432.CCR-18-1139 29950350

[22] You R Artichoker J Ray A Gonzalez Velozo H Rock DA Conner KP. Visualizing spatial and stoichiometric barriers to bispecific T-cell engager efficacy. Cancer Immunol Res. (2022) 10:698–712. doi: 10.1158/2326-6066.CIR-21-0594 35413104 PMC9177795

[23] Li J Stagg NJ Johnston J Harris MJ Menzies SA DiCara D. Membrane-proximal epitope facilitates efficient T cell synapse formation by anti-fcRH5/CD3 and is a requirement for myeloma cell killing. Cancer Cell. (2017) 31:383–95. doi: 10.1016/j.ccell.2017.02.001 PMC5357723 28262555

[24] Prokhnevska N Cardenas MA Valanparambil RM Sobierajska E Barwick BG Jansen C. CD8(+) T cell activation in cancer comprises an initial activation phase in lymph nodes followed by effector differentiation within the tumor. Immunity. (2023) 56:107–24.e5. doi: 10.1016/j.immuni.2022.12.002 36580918 PMC10266440

[25] Ma L Dichwalkar T Chang JYH Cossette B Garafola D Zhang AQ. Enhanced CAR-T cell activity against solid tumors by vaccine boosting through the chimeric receptor. Science. (2019) 365:162–8. doi: 10.1126/science.aav8692 PMC6800571 31296767

[26] Reinhard K Rengstl B Oehm P Michel K Billmeier A Hayduk N. An RNA vaccine drives expansion and efficacy of claudin-CAR-T cells against solid tumors. Science. (2020) 367:446–53. doi: 10.1126/science.aay5967 31896660

[27] Wang X Diamond DJ Forman SJ Nakamura R. Development of CMV-CD19 bi-specific CAR T cells with post-infusion *in vivo* boost using an anti-CMV vaccine. Int J Hematol. (2021) 114:544–53. doi: 10.1007/s12185-021-03215-6 PMC8475363 34561840

[28] Groeneveldt C Kinderman P van den Wollenberg DJM van den Oever RL Middelburg J Mustafa DAM. Preconditioning of the tumor microenvironment with oncolytic reovirus converts CD3-bispecific antibody treatment into effective immunotherapy. J Immunother Cancer. (2020) 8:e001191. doi: 10.1136/jitc-2020-001191 33082167 PMC7577070

[29] Wang Q Ma X Wu H Zhao C Chen J Li R. Oncolytic adenovirus with MUC16-BiTE shows enhanced antitumor immune response by reversing the tumor microenvironment in PDX model of ovarian cancer. Oncoimmunology. (2022) 11:2096362. doi: 10.1080/2162402X.2022.2096362 35800156 PMC9255048

[30] Petersen CT Hassan M Morris AB Jeffery J Lee K Jagirdar N. Improving T-cell expansion and function for adoptive T-cell therapy using ex vivo treatment with PI3Kdelta inhibitors and VIP antagonists. Blood Adv. (2018) 2:210–23. doi: 10.1182/bloodadvances.2017011254 PMC5812323 29386194

[31] Verma R Foster RE Horgan K Mounsey K Nixon H Smalle N. Lymphocyte depletion and repopulation after chemotherapy for primary breast cancer. Breast Cancer Res. (2016) 18:10. doi: 10.1186/s13058-015-0669-x 26810608 PMC4727393

[32] Cortes-Selva D Perova T Skerget S Vishwamitra D Stein S Boominathan R. Correlation of immune fitness with response to teclistamab in relapsed/refractory multiple myeloma in the MajesTEC-1 study. Blood. (2024) 144:615–28. doi: 10.1182/blood.2023022823 PMC11347796 38657201

[33] Hipp S Voynov V Drobits-Handl B Giragossian C Trapani F Nixon AE. A bispecific DLL3/CD3 igG-like T-cell engaging antibody induces antitumor responses in small cell lung cancer. Clin Cancer Res. (2020) 26:5258–68. doi: 10.1158/1078-0432.CCR-20-0926 32554516

[34] Kohnke T Krupka C Tischer J Knosel T Subklewe M. Increase of PD-L1 expressing B-precursor ALL cells in a patient resistant to the CD19/CD3-bispecific T cell engager antibody blinatumomab. J Hematol Oncol. (2015) 8:111. doi: 10.1186/s13045-015-0213-6 26449653 PMC4599591

[35] Zhu WM Middleton MR. Combination therapies for the optimisation of Bispecific T-cell Engagers in cancer treatment. Immunother Adv. (2023) 3:ltad013. doi: 10.1093/immadv/ltad013 37599903 PMC10439528

[36] Chang CH Wang Y Li R Rossi DL Liu D Rossi EA. Combination therapy with bispecific antibodies and PD-1 blockade enhances the antitumor potency of T cells. Cancer Res. (2017) 77:5384–94. doi: 10.1158/0008-5472.CAN-16-3431 28819027

[37] Crawford A Haber L Kelly MP Vazzana K Canova L Ram P. A Mucin 16 bispecific T cell-engaging antibody for the treatment of ovarian cancer. Sci Transl Med. (2019) 11:e7534. doi: 10.1126/scitranslmed.aau7534 31217340

[38] Sam J Colombetti S Fauti T Roller A Biehl M Fahrni L. Combination of T-cell bispecific antibodies with PD-L1 checkpoint inhibition elicits superior anti-tumor activity. Front Oncol. (2020) 10:575737. doi: 10.3389/fonc.2020.575737 33330050 PMC7735156

[39] Grosser R Cherkassky L Chintala N Adusumilli PS. Combination immunotherapy with CAR T cells and checkpoint blockade for the treatment of solid tumors. Cancer Cell. (2019) 36:471–82. doi: 10.1016/j.ccell.2019.09.006 PMC7171534 31715131

[40] Imai C Mihara K Andreansky M Nicholson IC Pui CH Geiger TL. Chimeric receptors with 4-1BB signaling capacity provoke potent cytotoxicity against acute lymphoblastic leukemia. Leukemia. (2004) 18:676–84. doi: 10.1038/sj.leu.2403302 14961035

[41] Savoldo B Ramos CA Liu E Mims MP Keating MJ Carrum G. CD28 costimulation improves expansion and persistence of chimeric antigen receptor-modified T cells in lymphoma patients. J Clin Invest. (2011) 121:1822–6. doi: 10.1172/JCI46110 PMC3083795 21540550

[42] Chiu D Tavaré R Haber L Aina OH Vazzana K Ram P. A PSMA-targeting CD3 bispecific antibody induces antitumor responses that are enhanced by 4-1BB costimulation. Cancer Immunol Res. (2020) 8:596–608. doi: 10.1158/2326-6066.CIR-19-0518 32184296

[43] Correnti CE Laszlo GS de van der Schueren WJ Godwin CD Bandaranayake A Busch MA. Simultaneous multiple interaction T-cell engaging (SMITE) bispecific antibodies overcome bispecific T-cell engager (BiTE) resistance via CD28 co-stimulation. Leukemia. (2018) 32:1239–43. doi: 10.1038/s41375-018-0014-3 PMC5943151 29588544

[44] Skokos D Waite JC Haber L Crawford A Hermann A Ullman E. A class of costimulatory CD28-bispecific antibodies that enhance the antitumor activity of CD3-bispecific antibodies. Sci Transl Med. (2020) 12:e7888. doi: 10.1126/scitranslmed.aaw7888 31915305

[45] Middelburg J Schaap G Sluijter M Lloyd K Ovcinnikovs V Schuurman J. Cancer vaccines compensate for the insufficient induction of protective tumor-specific immunity of CD3 bispecific antibody therapy. J ImmunoTherapy Cancer. (2025) 13:e010331. doi: 10.1136/jitc-2024-010331 PMC11749218 39800374

[46] Brouwer-Visser J Fiaschi N Deering RP Cygan KJ Scott D Jeong S. Molecular assessment of intratumoral immune cell subsets and potential mechanisms of resistance to odronextamab, a CD20×CD3 bispecific antibody, in patients with relapsed/refractory B-cell non-Hodgkin lymphoma. J ImmunoTherapy Cancer. (2024) 12:e008338. doi: 10.1136/jitc-2023-008338 PMC10961523 38519055

[47] Schuster SJ Huw LY Bolen CR Maximov V Polson AG Hatzi K. Loss of CD20 expression as a mechanism of resistance to mosunetuzumab in relapsed/refractory B-cell lymphomas. Blood. (2024) 143:822–32. doi: 10.1182/blood.2023022348 PMC10934296 38048694

[48] AldossISongJStillerTNguyenTPalmerJO’DonnellM. Correlates of resistance and relapse during blinatumomab therapy for relapsed/refractory acute lymphoblastic leukemia. Am J Hematol. (2017) 92:858–65. doi: 10.1002/ajh.24783 28494518

[49] Gonzalez-Exposito R Semiannikova M Griffiths B Khan K Barber LJ Woolston A. CEA expression heterogeneity and plasticity confer resistance to the CEA-targeting bispecific immunotherapy antibody cibisatamab (CEA-TCB) in patient-derived colorectal cancer organoids. J Immunother Cancer. (2019) 7:101. doi: 10.1186/s40425-019-0575-3 30982469 PMC6463631

[50] Jacoby E. Relapse and resistance to CAR-T cells and blinatumomab in hematologic Malignancies. Clin Hematol Int. (2019) 1:79–84. doi: 10.2991/chi.d.190219.001 34595414 PMC8432394

[51] Frerichs KA Broekmans MEC Marin Soto JA van Kessel B Heymans MW Holthof LC. Preclinical activity of JNJ-7957, a novel BCMA×CD3 bispecific antibody for the treatment of multiple myeloma, is potentiated by daratumumab. Clin Cancer Res. (2020) 26:2203–15. doi: 10.1158/1078-0432.CCR-19-2299 31969333

[52] Hegde M Mukherjee M Grada Z Pignata A Landi D Navai SA. Tandem CAR T cells targeting HER2 and IL13Rα2 mitigate tumor antigen escape. J Clin Invest. (2021) 131:e152477. doi: 10.1172/JCI152477 34196303 PMC8245163

[53] Chen DS Mellman I. Oncology meets immunology: the cancer-immunity cycle. Immunity. (2013) 39:1–10. doi: 10.1016/j.immuni.2013.07.012 23890059

[54] Benonisson H Altıntaş I Sluijter M Verploegen S Labrijn AF Schuurhuis DH. CD3-Bispecific Antibody Therapy Turns Solid Tumors into Inflammatory Sites but Does Not Install Protective Memory. Mol Cancer Ther. (2019) 18:312–22. doi: 10.1158/1535-7163.MCT-18-0679 30381448

[55] Rojas LA Sethna Z Soares KC Olcese C Pang N Patterson E. Personalized RNA neoantigen vaccines stimulate T cells in pancreatic cancer. Nature. (2023) 618:144–50. doi: 10.1038/s41586-023-06063-y PMC10171177 37165196

[56] Russell SJ Barber GN. Oncolytic viruses as antigen-agnostic cancer vaccines. Cancer Cell. (2018) 33:599–605. doi: 10.1016/j.ccell.2018.03.011 29634947 PMC5918693

[57] Bruni D Angell HK Galon J. The immune contexture and Immunoscore in cancer prognosis and therapeutic efficacy. Nat Rev Cancer. (2020) 20:662–80. doi: 10.1038/s41568-020-0285-7 32753728

[58] Fridman WH Pagès F Sautès-Fridman C Galon J. The immune contexture in human tumours: impact on clinical outcome. Nat Rev Cancer. (2012) 12:298–306. doi: 10.1038/nrc3245 22419253

[59] Ardighieri L Missale F Bugatti M Gatta LB Pezzali I Monti M. Infiltration by CXCL10 secreting macrophages is associated with antitumor immunity and response to therapy in ovarian cancer subtypes. Front Immunol. (2021) 12:690201. doi: 10.3389/fimmu.2021.690201 34220848 PMC8253056

[60] Gungabeesoon J Gort-Freitas NA Kiss M Bolli E Messemaker M Siwicki M. A neutrophil response linked to tumor control in immunotherapy. Cell. (2023) 186:1448–64.e20. doi: 10.1016/j.cell.2023.02.032 37001504 PMC10132778

[61] Besla R Penuel E Del Rosario G Cosino E Myrta S Dillon M. T cell-dependent bispecific therapy enhances innate immune activation and antibody-mediated killing. Cancer Immunol Res. (2024) 12:60–71. doi: 10.1158/2326-6066.CIR-23-0072 37902604

[62] Mulder K Patel AA Kong WT Piot C Halitzki E Dunsmore G. Cross-tissue single-cell landscape of human monocytes and macrophages in health and disease. Immunity. (2021) 54:1883–900.e5. doi: 10.1016/j.immuni.2021.07.007 34331874

[63] Wang MM Coupland SE Aittokallio T Figueiredo CR. Resistance to immune checkpoint therapies by tumour-induced T-cell desertification and exclusion: key mechanisms, prognostication and new therapeutic opportunities. Br J Cancer. (2023) 129:1212–24. doi: 10.1038/s41416-023-02361-4 PMC10575907 37454231

[64] Blomberg OS Spagnuolo L Garner H Voorwerk L Isaeva OI van Dyk E. IL-5-producing CD4(+) T cells and eosinophils cooperate to enhance response to immune checkpoint blockade in breast cancer. Cancer Cell. (2023) 41:106–23.e10. doi: 10.1016/j.ccell.2022.11.014 36525971

[65] Hu A Sun L Lin H Liao Y Yang H Mao Y. Harnessing innate immune pathways for therapeutic advancement in cancer. Signal Transduct Target Ther. (2024) 9:68. doi: 10.1038/s41392-024-01765-9 38523155 PMC10961329

[66] Ackerman SE Pearson CI Gregorio JD Gonzalez JC Kenkel JA Hartmann FJ. Immune-stimulating antibody conjugates elicit robust myeloid activation and durable antitumor immunity. Nat Cancer. (2021) 2:18–33. doi: 10.1038/s43018-020-00136-x 35121890 PMC9012298

[67] Tang L Pan S Wei X Xu X Wei Q. Arming CAR-T cells with cytokines and more: Innovations in the fourth-generation CAR-T development. Mol Ther. (2023) 31:3146–62. doi: 10.1016/j.ymthe.2023.09.021 PMC10638038 37803832

[68] Li J Slaga D Johnston J Junttila TT. IMiDs augment CD3-bispecific antibody-induced CD8+ T-cell cytotoxicity and expansion by enhancing IL2 production. Mol Cancer Ther. (2023) 22:659–66. doi: 10.1158/1535-7163.MCT-22-0498 PMC10157361 36822576

[69] Xu H Buhtoiarov IN Guo H Cheung NV. A novel multimeric IL15/IL15Rα-Fc complex to enhance cancer immunotherapy. Oncoimmunology. (2021) 10:1893500. doi: 10.1080/2162402X.2021.1893500 33763293 PMC7954438

[70] Schmohl JU Felices M Taras E Miller JS Vallera DA. Enhanced ADCC and NK cell activation of an anticarcinoma bispecific antibody by genetic insertion of a modified IL-15 cross-linker. Mol Ther. (2016) 24:1312–22. doi: 10.1038/mt.2016.88 PMC5088765 27157665

[71] FDA. FDA approves nogapendekin alfa inbakicept-pmln for BCG-unresponsive non-muscle invasive bladder cancer. Available online at: https://www.fda.gov/drugs/resources-information-approved-drugs/fda-approves-nogapendekin-alfa-inbakicept-pmln-bcg-unresponsive-non-muscle-invasive-bladder-cancer2024 (Accessed December 17, 2024).

[72] Hua YJ Liu YL Wen K Kurts C Wu H Mei Q. Potentially improved response of COVID-19 vaccinated nasopharyngeal cancer patients to combination therapy with anti-PD-1 blockade and chemotherapy. Ann Oncol. (2023) 34:121–3. doi: 10.1016/j.annonc.2022.10.002 PMC9540702 36216192

[73] Qian Y Zhu Z Mo Y-Y Zhang Z. COVID-19 vaccination is associated with enhanced efficacy of anti-PD-(L)1 immunotherapy in advanced NSCLC patients: a real-world study. Infect Agents Cancer. (2023) 18:50. doi: 10.1186/s13027-023-00526-7 PMC10485982 37679851

[74] Lopez-Olivo MA Valerio V Karpes Matusevich AR Brizio M Kwok M Geng Y. Safety and efficacy of influenza vaccination in patients receiving immune checkpoint inhibitors. Systematic review with meta-analysis. Vaccines (Basel). (2022) 10:1195. doi: 10.3390/vaccines10081195 36016085 PMC9412390

[75] Audisio A Buttigliero C Delcuratolo MD Parlagreco E Audisio M Ungaro A. New perspectives in the medical treatment of non-muscle-invasive bladder cancer: immune checkpoint inhibitors and beyond. Cells. (2022) 11:357. doi: 10.3390/cells11030357 35159167 PMC8834622

[76] Cheever MA Higano CS. PROVENGE (Sipuleucel-T) in prostate cancer: the first FDA-approved therapeutic cancer vaccine. Clin Cancer Res. (2011) 17:3520–6. doi: 10.1158/1078-0432.CCR-10-3126 21471425

[77] Pol J Kroemer G Galluzzi L. First oncolytic virus approved for melanoma immunotherapy. OncoImmunology. (2016) 5:e1115641. doi: 10.1080/2162402X.2015.1115641 26942095 PMC4760283

[78] Sahin U Oehm P Derhovanessian E Jabulowsky RA Vormehr M Gold M. An RNA vaccine drives immunity in checkpoint-inhibitor-treated melanoma. Nature. (2020) 585:107–12. doi: 10.1038/s41586-020-2537-9 32728218

[79] Weber JS Carlino MS Khattak A Meniawy T Ansstas G Taylor MH. Individualised neoantigen therapy mRNA-4157 (V940) plus pembrolizumab versus pembrolizumab monotherapy in resected melanoma (KEYNOTE-942): a randomised, phase 2b study. Lancet. (2024) 403:632–44. doi: 10.1016/S0140-6736(23)02268-7 38246194

[80] Sheth SS Oh JE Bellone S Siegel ER Greenman M Mutlu L. Randomized Phase II Trial of Imiquimod with or without 9-Valent HPV Vaccine versus Observation in Patients with High-grade Pre-neoplastic Cervical Lesions (NCT02864147). Clin Cancer Res. (2024) 30:1768–77. doi: 10.1158/1078-0432.CCR-23-3639 38592381

[81] Arcangeli S Bove C Mezzanotte C Camisa B Falcone L Manfredi F. CAR T cell manufacturing from naive/stem memory T lymphocytes enhances antitumor responses while curtailing cytokine release syndrome. J Clin Invest. (2022) 132:e150807. doi: 10.1172/JCI150807 35503659 PMC9197529

[82] Mehta PH Fiorenza S Koldej RM Jaworowski A Ritchie DS Quinn KM. T cell fitness and autologous CAR T cell therapy in haematologic Malignancy. Front Immunol. (2021) 12:780442. doi: 10.3389/fimmu.2021.780442 34899742 PMC8658247

[83] Segal NH Melero I Moreno V Steeghs N Marabelle A Rohrberg K. CEA-CD3 bispecific antibody cibisatamab with or without atezolizumab in patients with CEA-positive solid tumours: results of two multi-institutional Phase 1 trials. Nat Commun. (2024) 15:4091. doi: 10.1038/s41467-024-48479-8 38750034 PMC11096172

[84] Dorff T Horvath LG Autio K Bernard-Tessier A Rettig MB Machiels JP. A phase I study of acapatamab, a half-life extended, PSMA-targeting bispecific T-cell engager for metastatic castration-resistant prostate cancer. Clin Cancer Res. (2024) 30:1488–500. doi: 10.1158/1078-0432.CCR-23-2978 PMC11395298 38300720

[85] Paz-Ares L Champiat S Lai WV Izumi H Govindan R Boyer M. Tarlatamab, a first-in-class DLL3-targeted bispecific T-cell engager, in recurrent small-cell lung cancer: an open-label, phase I study. J Clin Oncol. (2023) 41:2893–903. doi: 10.1200/JCO.22.02823 PMC10414718 36689692

[86] Ball K Dovedi SJ Vajjah P Phipps A. Strategies for clinical dose optimization of T cell-engaging therapies in oncology. MAbs. (2023) 15:2181016. doi: 10.1080/19420862.2023.2181016 36823042 PMC9980545

