# Supplementary material for: Improving CD3 bispecific antibody therapy in solid tumors using combination strategies
Source: Front Oncol. 2025 Feb 10;15:1548446. doi: 10.3389/fonc.2025.1548446 (PMC11847677; doi:10.3389/fonc.2025.1548446)
Supplement: Supplementary file 1 [file Table1.pdf]

Supplementary Table – Overview of T-cell stimulating vaccines and innate activators, which could be potentially combined with CD3 bsAb in the clinic. The selection was based on the ability of vaccines to induce T cell response, irrespective of specificity.

Therapeutic type	Name	Components / targets	Administration route	Clinical phase	Indication	Company	Refs
**Live-attenuated vaccine**	Dengvaxia	Dengue serotype 1-4 proteins	Subcutaneous	Marketed	Dengue	Sanofi	(1)
	denvAX	Dengue serotypes 1-4	Subcutaneous	Marketed	Dengue	Takeda	(2)
	Bacille Calmette–Guérin (BCG)	Mycobacterium bovis	Percutaneous	Marketed	Tuberculosis	Merck	(3)
	ERVEBO	Ebola virus glycoprotein	Intramuscular	Marketed	Ebola	Merck	(4)
	Zabdeno & Mvabea	Ebola virus glycoprotein	Intramuscular	Marketed	Ebola	Janssen	(5)
	Flumist Quadrivalent	H1N1, H3N2, B/Victoria, B/ Phuket	Intranasal	Marketed	Influenza	MedImmune	(6)
**Inactivated virus**	Euvichol	Vibrio cholera O1 and O139	Oral	Marketed	Cholera	EuBiologics	(7)
	Dukoral	Vibrio cholera O1	Oral	Marketed	Cholera	Valneva	(8)
	HAVRIX	Hep A HM175	Intramuscular	Marketed	Hep A	GSK	(9)
	Healive	Hep A	Intramuscular	Marketed	Hep A	Sinovac	(9)
	Fluzone Quadrivalent	H1N1, H3N2, B/Victoria, B/ Yamagata	Intramuscular	Marketed	Influenza	Sanofi	(10)
	Fluad Quadrivalent	H1N1, H3N2, B/Victoria, B/ Yamagata	Intramuscular	Marketed	Influenza	CSL Seqirus	
	Flucelvax	H1N1, H3N2, B/Victoria, B/ Yamagata	Intramuscular	Marketed	Influenza	CSL Seqirus	(10)
**Protein-based vaccine**	Adacel	Tdap	Intramuscular	Marketed	Diphtheria, Tetanus, Pertussis	Sanofi	(11)
	Boostrix	Tdap	Intramuscular	Marketed	Diphtheria, Tetanus, Pertussis	GSK	(12)
	Infanrix-hexa	DTPa-HBV-IPV-Hib	Intramuscular	Marketed	Diphtheria, Tetanus, Pertussis, Hep B, polio, H. influenzae type B	GSK	(12)
	Gardasil	HPV type 6, 11, 16, 18	Intramuscular	Marketed	HPV	Merck	(13)

	Cervarix	HPV type 16,18	Intramuscular	Marketed	HPV	GSK	(14)
	Engerix-B	HBsAg	Intramuscular	Marketed	Hep B	GSK	(15)
	Flublock Quadrivalent	H1N1, H3N2, B/Victoria, B/ Yamagata	Intramuscular	Marketed	Influenza	Sanofi	(10)
**Adenovirus-based vaccines**	Jcovden	SARS-CoV-2 spike protein	Intramuscular	Marketed	Covid-19	Janssen	(16)
	Vaxzevria	SARS-CoV-2 spike protein	Intramuscular	Marketed	Covid-19	AstraZeneca	(17)
	Convidecia	SARS-CoV-2 spike protein	Intramuscular	Marketed	Covid-19	CanSino Biologics	(18)
	ChAd3-EBO-Z	Ebola virus glycoprotein	Intramuscular	Ph III	Ebola	GSK	(19)
	Ad26.RSV.preF	Prefusion F	Intramuscular	Ph III	RSV	Janssen	(20)
	T-VEC	GM-CSF	Intratumoral	Marketed	Melanoma	Amgen	(21)
**mRNA-based vaccines**	Spikevax	SARS-CoV-2 spike protein	Intramuscular	Marketed	Covid-19	Moderna	(22)
	mRNA-1273.815	SARS-CoV-2 spike protein	Intramuscular	Marketed	Covid-19	Moderna	(23)
	mRESVIA	RSV F glycoprotein	Intramuscular	Marketed	RSV	Moderna	(24)
	mRNA-1010	Hemagglutinin glycoprotein	Intramuscular	Ph III	Flu	Moderna	(25)
	mRNA-1647	Glycoprotein B pentamer	Intramuscular	Ph III	CMV	Moderna	(26)
	mRNA-4157/V940	34 patient-specific tumor neoantigens	Intramuscular	Ph II / III	Melanoma	Moderna/ Merck	(27)
	COMIRNATY	SARS-CoV-2 spike protein	Intramuscular	Marketed	Covid-19	BioNTech/ Pfizer	(28)
	BNT161	Hemagglutinin glycoprotein	Intramuscular	Ph III	Influenza	BioNTech	.
	BNT111	NY-ESO-1, MAGE-A3, TPTE, tyrosinase	Intravenous	Ph II	Melanoma	BioNTech	(29)
	BNT113	E6 & E7 proteins	Intravenous	Ph II	HPV16+ head & neck cancer	BioNTech	.
	BNT116	Hexavalent TAA	Intravenous	Ph II	NSCLC	BioNTech	(30)
	BNT122	20 patient-specific tumor neoantigens	Intravenous	Ph II	Solid tumors	BioNTech	(31)
**Cell-based vaccines**	Sipuleucel-T	Autologous PBMCs, Prostatic acid phosphatase, GM-CSF	Intraveneous	Marketed	Prostate cancer	Dendreon Pharmaceuticals	(32)
**ISAC**	BDC-1001	HER2, TLR7/8	Intravenously	Ph II	Solid tumors	Bolt Biotherapeutics	(33)
	TAK-500	CCR2, STING	Intravenously	Ph II	Solid tumors	Takeda	(34)

**Small molecule**	Imiquimod	TLR7	Topical	Marketed	Basal cell carcinoma	3M Pharmaceuticals	(35)

References

1. Hadinegoro SR, Arredondo-Garcia JL, Capeding MR, Deseda C, Chotpitayasunondh T, Dietze R, et al. Efficacy and Long-Term Safety of a Dengue Vaccine in Regions of Endemic Disease. N Engl J Med. 2015;373(13):1195-206.
2. Tricou V, Yu D, Reynales H, Biswal S, Saez-Llorens X, Sirivichayakul C, et al. Long-term efficacy and safety of a tetravalent dengue vaccine (TAK-003): 4·5-year results from a phase 3, randomised, double-blind, placebo-controlled trial. Lancet Glob Health. 2024;12(2):e257-e70.
3. Colditz GA, Brewer TF, Berkey CS, Wilson ME, Burdick E, Fineberg HV, et al. Efficacy of BCG vaccine in the prevention of tuberculosis. Meta-analysis of the published literature. Jama. 1994;271(9):698-702.
4. Dahlke C, Kasonta R, Lunemann S, Krähling V, Zinser ME, Biedenkopf N, et al. Dose-dependent T-cell Dynamics and Cytokine Cascade Following rVSV-ZEBOV Immunization. EBioMedicine. 2017;19:107-18.
5. Mutua G, Anzala O, Luhn K, Robinson C, Bockstal V, Anumendem D, et al. Safety and Immunogenicity of a 2-Dose Heterologous Vaccine Regimen With Ad26.ZEBOV and MVA-BN-Filo Ebola Vaccines: 12-Month Data From a Phase 1 Randomized Clinical Trial in Nairobi, Kenya. J Infect Dis. 2019;220(1):57-67.
6. Basha S, Hazenfeld S, Brady RC, Subbramanian RA. Comparison of antibody and T-cell responses elicited by licensed inactivated- and live-attenuated influenza vaccines against H3N2 hemagglutinin. Hum Immunol. 2011;72(6):463-9.
7. Song KR, Chapagain RH, Tamrakar D, Shrestha R, Kanodia P, Chaudhary S, et al. Safety and immunogenicity of the Euvichol-S oral cholera vaccine for prevention of Vibrio cholerae O1 infection in Nepal: an observer-blind, active-controlled, randomised, non-inferiority, phase 3 trial. Lancet Glob Health. 2024;12(5):e826-e37.
8. van Splunter M, van Hoffen E, Floris-Vollenbroek EG, Timmerman H, de Bos EL, Meijer B, et al. Oral cholera vaccination promotes homing of IgA(+) memory B cells to the large intestine and the respiratory tract. Mucosal Immunol. 2018;11(4):1254-64.
9. Schmidtke P, Habermehl P, Knuf M, Meyer CU, Sänger R, Zepp F. Cell mediated and antibody immune response to inactivated hepatitis A vaccine. Vaccine. 2005;23(44):5127-32.
10. Richards KA, Moritzky S, Shannon I, Fitzgerald T, Yang H, Branche A, et al. Recombinant HA-based vaccine outperforms split and subunit vaccines in elicitation of influenza-specific CD4 T cells and CD4 T cell-dependent antibody responses in humans. NPJ Vaccines. 2020;5:77.
11. Blatter M, Friedland LR, Weston WM, Li P, Howe B. Immunogenicity and safety of a tetanus toxoid, reduced diphtheria toxoid and three-component acellular pertussis vaccine in adults 19-64 years of age. Vaccine. 2009;27(5):765-72.
12. van der Lee S, van Rooijen DM, de Zeeuw-Brouwer ML, Bogaard MJM, van Gageldonk PGM, Marinovic AB, et al. Robust Humoral and Cellular Immune Responses to Pertussis in Adults After a First Acellular Booster Vaccination. Front Immunol. 2018;9:681.

13. Pasmans H, Berkowska MA, Diks AM, de Mooij B, Groenland RJ, de Rond L, et al. Characterization of the early cellular immune response induced by HPV vaccines. Front Immunol. 2022;13:863164.
14. Einstein MH, Levin MJ, Chatterjee A, Chakhtoura N, Takacs P, Catteau G, et al. Comparative humoral and cellular immunogenicity and safety of human papillomavirus (HPV)-16/18 AS04-adjuvanted vaccine and HPV-6/11/16/18 vaccine in healthy women aged 18-45 years: follow-up through Month 48 in a Phase III randomized study. Hum Vaccin Immunother. 2014;10(12):3455-65.
15. Elias G, Meysman P, Bartholomeus E, De Neuter N, Keersmaekers N, Suls A, et al. Preexisting memory CD4 T cells in naïve individuals confer robust immunity upon hepatitis B vaccination. Elife. 2022;11:e68388.
16. Cárdenas V, Le Gars M, Truyers C, Ruiz-Guiñazú J, Struyf F, Colfer A, et al. Safety and immunogenicity of Ad26.COV2.S in adults: A randomised, double-blind, placebo-controlled Phase 2a dose-finding study. Vaccine. 2024;42(16):3536-46.
17. Ewer KJ, Barrett JR, Belij-Rammerstorfer S, Sharpe H, Makinson R, Morter R, et al. T cell and antibody responses induced by a single dose of ChAdOx1 nCoV-19 (AZD1222) vaccine in a phase 1/2 clinical trial. Nat Med. 2021;27(2):270-8.
18. Zhu FC, Guan XH, Li YH, Huang JY, Jiang T, Hou LH, et al. Immunogenicity and safety of a recombinant adenovirus type-5-vectored COVID-19 vaccine in healthy adults aged 18 years or older: a randomised, double-blind, placebo-controlled, phase 2 trial. Lancet. 2020;396(10249):479-88.
19. Happe M, Hofstetter AR, Wang J, Yamshchikov GV, Holman LA, Novik L, et al. Heterologous cAd3-Ebola and MVA-EbolaZ vaccines are safe and immunogenic in US and Uganda phase 1/1b trials. NPJ Vaccines. 2024;9(1):67.
20. Williams K, Bastian AR, Feldman RA, Omoruyi E, de Paepe E, Hendriks J, et al. Phase 1 Safety and Immunogenicity Study of a Respiratory Syncytial Virus Vaccine With an Adenovirus 26 Vector Encoding Prefusion F (Ad26.RSV.preF) in Adults Aged ≥60 Years. J Infect Dis. 2020;222(6):979-88.
21. Malvehy J, Samoylenko I, Schadendorf D, Gutzmer R, Grob JJ, Sacco JJ, et al. Talimogene laherparepvec upregulates immune-cell populations in non-injected lesions: findings from a phase II, multicenter, open-label study in patients with stage IIIB-IVM1c melanoma. J Immunother Cancer. 2021;9(3):e001621.
22. Baden LR, El Sahly HM, Essink B, Kotloff K, Frey S, Novak R, et al. Efficacy and Safety of the mRNA-1273 SARS-CoV-2 Vaccine. N Engl J Med. 2021;384(5):403-16.
23. Chalkias S, McGhee N, Whatley JL, Essink B, Brosz A, Tomassini JE, et al. Interim Report of the Reactogenicity and Immunogenicity of Severe Acute Respiratory Syndrome Coronavirus 2 XBB-Containing Vaccines. J Infect Dis. 2024;230(2):e279-e86.
24. Wilson E, Goswami J, Baqui AH, Doreski PA, Perez-Marc G, Zaman K, et al. Efficacy and Safety of an mRNA-Based RSV PreF Vaccine in Older Adults. N Engl J Med. 2023;389(24):2233-44.
25. Ananworanich J, Lee IT, Ensz D, Carmona L, Schaefers K, Avanesov A, et al. Safety and Immunogenicity of mRNA-1010, an Investigational Seasonal Influenza Vaccine, in Healthy Adults: Final Results From a Phase 1/2 Randomized Trial. J Infect Dis. 2024:3631.
26. Wu K, Hou YJ, Makrinos D, Liu R, Zhu A, Koch M, et al. Characterization of humoral and cellular immunologic responses to an mRNA-based human cytomegalovirus vaccine from a phase 1 trial of healthy adults. J Virol. 2024;98(4):e0160323.
27. Weber JS, Carlino MS, Khattak A, Meniawy T, Ansstas G, Taylor MH, et al. Individualised neoantigen therapy mRNA-4157 (V940) plus pembrolizumab versus pembrolizumab monotherapy in resected melanoma (KEYNOTE-942): a randomised, phase 2b study. Lancet. 2024;403(10427):632-44.

28. Polack FP, Thomas SJ, Kitchin N, Absalon J, Gurtman A, Lockhart S, et al. Safety and Efficacy of the BNT162b2 mRNA Covid-19 Vaccine. N Engl J Med. 2020;383(27):2603-15.
29. Sahin U, Oehm P, Derhovanessian E, Jabulowsky RA, Vormehr M, Gold M, et al. An RNA vaccine drives immunity in checkpoint-inhibitor-treated melanoma. Nature. 2020;585(7823):107-12.
30. Deme D, Öven B, Göker E, Lang I, Brück P, Wenger M, et al. 597 Preliminary results from LuCa-MERIT-1, a first-in-human Phase I trial evaluating the fixed antigen RNA vaccine BNT116 in patients with advanced non-small cell lung cancer. Journal for ImmunoTherapy of Cancer. 2023;11(Suppl 1):A679-A.
31. Rojas LA, Sethna Z, Soares KC, Olcese C, Pang N, Patterson E, et al. Personalized RNA neoantigen vaccines stimulate T cells in pancreatic cancer. Nature. 2023;618(7963):144-50.
32. Antonarakis ES, Small EJ, Petrylak DP, Quinn DI, Kibel AS, Chang NN, et al. Antigen-Specific CD8 Lytic Phenotype Induced by Sipuleucel-T in Hormone-Sensitive or Castration-Resistant Prostate Cancer and Association with Overall Survival. Clin Cancer Res. 2018;24(19):4662-71.
33. Li BT, Pegram MD, Lee K-W, Sharma M, Lee J, Spira AI, et al. A phase 1/2 study of a first-in-human immune-stimulating antibody conjugate (ISAC) BDC-1001 in patients with advanced HER2-expressing solid tumors. Journal of Clinical Oncology. 2023;41(16_suppl):2538-2538.
34. Singh H, Diamond JR, Henry JT, Olszanski AJ, Rasco D, Patel SP, et al. 1077TiP TAK-500 as a single agent and in combination with pembrolizumab in patients (pts) with advanced solid tumors: Rationale and design of a phase I/II study. Annals of Oncology. 2023;34:S648-S9.
35. Schon M, Schon MP. The antitumoral mode of action of imiquimod and other imidazoquinolines. Curr Med Chem. 2007;14(6):681-7.
